# A closer look into the affect dynamics of adolescents with depression and the interactions with their parents: An ecological momentary assessment study

**DOI:** 10.1007/s00787-024-02447-1

**Published:** 2024-05-18

**Authors:** Loes H.C. Janssen, Bart Verkuil, Lisanne A.E.M. van Houtum, Mirjam C.M. Wever, Wilma G.M. Wentholt, Bernet M. Elzinga

**Affiliations:** 1 Department of Clinical Psychology, Faculty of Social and Behavioral Science, Leiden University, Wassenaarseweg 52, Leiden, AK 2333 Netherlands; 2 Leiden Institute for Brain and Cognition (LIBC), Leiden, Netherlands

**Keywords:** Adolescents, Depression, Experience sampling method, Parenting, Parent-child interactions

## Abstract

Adolescents with depression tend to perceive behavior of parents as less positive than adolescents without depression, but conclusions are based on retrospective reports assessed once or over long time intervals, with the risk of memory biases affecting the recall. The current study used ecological momentary assessments to examine the link between adolescent affect and the amount of warmth and criticism expressed by both mothers and fathers in families with adolescents with depression versus adolescents without psychopathology in daily life. It also explored the possible bias by assessing parenting on the momentary, daily (EMA), and retrospective level. The sample consisted of 34 adolescents with depression and 58 parents and 80 healthy controls and 151 parents (adolescents: *M*_*age*_ = 15.8, *SD* = 1.41; 67.5% girls, parents: *M*_*age*_ = 49.3, *SD* = 5.73; 54.1% mothers). Participants completed retrospective questionnaires and four surveys a day for 14 consecutive days. Preregistered multilevel models showed that momentary parenting reports of adolescents with depression and healthy controls did not differ. The associations between perceived parenting of both mothers and fathers and adolescent affect did also not differ between the two groups. These results illustrate that adolescents generally benefit from supportive parenting, but substantial differences between individuals were found. In contrast to the momentary data, both adolescents with depression and their parents did report more negative parenting on retrospective questionnaires than healthy controls and their parents indicating that adolescents with depression may have a negativity bias in their retrospective recall. These findings are highly relevant for clinical practice and underscore the need for careful assessments on different time scales and including all family members.

**Supplementary Information:**

The online version contains supplementary material available at 10.1007/s00787-024-02447-1.

The prevalence of mood disorders increases substantially during adolescence [1] and an early onset has been associated with higher recurrence rates [e.g., 2] and adverse psychosocial outcomes in adulthood [e.g., 3]. One of the key interpersonal factors that affects adolescent well-being is the relationship with parents [4]. Findings based on observational and retrospective self-report studies showed that a lack of warmth and critical parenting are related to depression in adolescents, but also that adolescent depression can impact parenting [5–7]. These studies, however, mainly focused on retrospective reports on parenting and may involve recall bias [8] which may be amplified for adolescents with depression [9]. Moreover, these studies focused on how families differed in their levels of parenting (between-family level), while parenting can change from day to day, and each family is unique with distinct parent-adolescent dynamics [10, 11]. Zooming in to the daily and moment-to-moment experiences of parent-adolescent interactions and the associations with the adolescent affect can provide valuable insights for clinical interventions. By using ecological momentary assessment [EMA; 12] we aimed to examine the link between parental warmth and criticism of mothers and fathers and adolescent affect in families with adolescents with depression (i.e., a current major depressive disorder (MDD) or dysthymia; DEP) and families with adolescents without psychopathology (i.e., healthy controls; HC). Additionally, we explored the possible impact of recall bias on parenting reports by assessing parenting on the momentary, daily level (both based on EMA), and a more global, retrospective level (using questionnaires).

Parent-adolescent interactions characterized by lack of warmth and support and elevated levels of conflict and criticism have been consistently linked to depression in adolescents [e.g., 5, 6, 7, 13] and depression later in life [14–16]. Most of this evidence, however, is based on retrospective self-report questionnaires assessed once or spanning large time intervals (e.g., last year). To overcome potential reporter bias, observational studies have been used to gain insights into the more fine-grained dynamics between parents and adolescents. These studies show that low levels of parental positivity relate to depression in children and adolescents, but findings are more mixed, probably also due to the variety of tasks and coding systems [see for review 17]. Still, an important limitation here is that it does not capture the daily life dynamics in a natural context in which parent-adolescent interactions occur [8]. Recent studies have shown that parenting is highly dependent on the context and can fluctuate over time (i.e., hours or days) within a family or person [18–20], but research addressing these everyday family dynamics in clinical samples of adolescents is limited.

To date, 12 EMA studies investigated affect in adolescents with a clinical depression [see review 21]. Some studies found that adolescents with mood disorders report lower levels of positive affect and higher levels of negative affect than healthy controls [22, 23], but others did not find differences in affect between depressed and non-depressed adolescents [24–26]. While EMA enables assessing the naturalistic dynamic setting of adolescents’ daily life, only three studies examined the social context (i.e., amount of time spent together or co-rumination with peers or family) of adolescents with depression [23, 27, 28]. Quality of time spent together with parents was, however, not assessed. This is an important omission, given the importance of parenting for adolescent well-being [e.g., 5, 6]. As a next step, we therefore examined whether momentary positive and negative affect as well as momentary parental warmth and criticism (from both adolescents’ and parents’ perspective) during parent-adolescent interactions differed between families with a DEP adolescent and families with a HC adolescent. Moreover, we compared parenting reports between families with DEP adolescents and HCs on different time scales: momentary, daily, and retrospective, to explore whether a recall bias [8] may influence parenting reports.

Previous studies in community samples have shown that on moments or days when adolescents perceive more parental warmth and less parental conflict, they report less negative affect and more positive affect [29–31]. Moreover, the strength of this association may differ between adolescents explained by depressive symptoms. For adolescents who reported more depressive symptoms, stronger associations were found between (a lack of) daily parental support and conflict and adolescent negative affect compared to adolescents who reported less depressive symptoms [29, 30]. On the momentary level, depressive symptoms only explained differences in associations over time [31]. For instance, adolescents with more depressive symptoms experienced a stronger increase in positive affect after a warm interaction with their parent. Although these studies provided some first insights, it is essential to include a clinical sample to investigate whether this also holds in adolescents with clinical depression, which is important for the ultimate goal of guiding clinical practice.

This preregistered study (https://osf.io/qjyp5) aimed to (1) examine whether adolescent momentary positive and negative affect and momentary parental warmth and criticism during parent-adolescent interactions differed between families with a DEP adolescent and HCs, (2) assess the within-person momentary association between perceived parenting behavior and affect during parent-child interactions, and (3) examine whether this association is stronger for DEP adolescents. We hypothesized that: 1a) DEP adolescents report less momentary positive and negative affect than HCs; 1b) DEP adolescents and (1c) their mothers and fathers report less parental warmth and more parental criticism during momentary parent-child interactions than HCs; 2) more perceived parental warmth and less perceived parental criticism of mothers and fathers at a given moment is associated with more positive and less negative affect at the same moment; 3) the associations between perceived parenting of mothers and fathers and adolescent affect during momentary parent-adolescent interactions are stronger for DEP adolescents compared to HCs. After preregistration, we added an exploratory aim: comparing parenting reports on the momentary, daily level (both based on EMA), and retrospective level (questionnaires) of families with DEP adolescents and HCs.

## Methods

### Sample

Data were used from RE-PAIR (Relations and Emotions in Parent Adolescent Interaction Research), which examines parent-adolescent interactions and adolescent mental well-being by comparing DEP adolescents and their parents to HC adolescents and their parents. The RE-PAIR study consisted of four parts: online questionnaires, a research day at the lab, two weeks of EMA, and an Magnetic Resonance Imaging (MRI)-scan session with the adolescent and one parent. The current study focused on the EMA part of RE-PAIR and also included several online questionnaires. The RE-PAIR study was approved by the Medical Ethics Review Committee (METC) of Leiden University Medical Centre (LUMC; research protocol: P17.241).

Families were included in the study if adolescents: were aged between 11 and 17 years when screened for psychopathology, started secondary school, lived with at least one primary caregiver who wanted to participate, and had a good command of the Dutch language. Participation with two parents – if possible – was preferred but this was no requirement. DEP adolescents had to meet criteria for a current MDD or dysthymia as primary diagnosis, and no other primary (neuro)psychiatric disorder or comorbid psychosis, substance use disorder or mental retardation. For HC adolescents, a lifetime MDD/dysthymia diagnosis, a current mental disorder, or a history of psychopathology in the last two years were exclusion criteria. Adolescent psychopathology was assessed with the Kiddie-Schedule for Affective Disorders and Schizophrenia – Present and Lifetime Version [K-SADS-PL; 32]. All participants signed informed consent. If adolescents were younger than 16 years of age, parents with legal custody also signed informed consent for the adolescent. For detailed information on sample recruitment and study procedure see Appendix 1.

In total, 114 families participated in the EMA of RE-PAIR: 80 HCs and their 151 parents, and 34 DEP adolescents and their 58 parents. Current primary diagnosis was MDD for 28 adolescents (82.4%) and dysthymia for 6 adolescents (17.6%). See Appendix 2 for comorbidity of adolescents and psychopathology of parents. Due to a branching error in questionnaires of one HC adolescent, we excluded that family resulting in a final sample of 79 HCs and 149 parents. Table 1 provides sample demographics. The majority of adolescents (96.3% HCs; 91.2% DEP adolescents) and parents (94.6% parents of HCs; 82.8% parents of DEP adolescents) were born in the Netherlands.

**Table 1:** Sample demographics and descriptive statistics

	HC		DEP		Difference^a^
	*N/obs*		*N/obs*		*p*
*Adolescents*					
Sex, % Female, (*n)*	79	63.3 (50)	34	76.5 (26)	
Age (years), *M(SD)*	79	15.9 (1.33)	34	15.7 (1.53)	
Highest level of education, % (*n*)	79		34		
Vocational		12.7 (10)		17.6 (6)	
Advanced secondary		32.9 (26)		23.5 (8)	
Pre-university		45.6 (36)		38.2 (13)	
Secondary vocational		6.3 (5)		14.7 (5)	
Higher professional		2.5 (2)		5.9 (2)	
Depressive symptoms (PHQ-9) *M* (SD)	79	4.77 (2.81)	34	20.21 (4.56)	< 0.001
CTQ					
Emotional abuse *M*(SD)	78	6.44 (2.27)	34	8.68 (3.72)	< 0.001
Emotional neglect *M*(SD)	78	7.94 (2.98)	34	10.94 (3.46)	< 0.001
PBI					
Care-mother *M*(SD)	78	31.91 (4.21)	34	27.03 (6.68)	< 0.001
Overprotection-mother *M*(SD)	78	3.51 (2.28)	34	5.88 (3.52)	< 0.001
Lack of autonomy-mother *M*(SD)	78	3.59 (2.87)	34	4.71 (4.37)	0.362
Care-father *M*(SD)	70	29.99 (5.17)	25	26.16 (6.10)	0.004
Overprotection-father *M*(SD)	70	3.06 (2.33)	25	5.32 (3.48)	< 0.001
Lack of autonomy-father *M*(SD)	70	3.59 (2.55)	25	4.92 (3.65)	0.141
*Daily level*					
Maternal warmth *M*(SD)	844	5.90 (1.04)	351	5.42 (1.47)	0.007
Maternal criticism *M*(SD)	844	2.01 (1.32)	351	1.97 (1.27)	0.698
Paternal warmth *M*(SD)	730	5.79 (1.20)	236	5.35 (1.44)	0.030
Paternal criticism *M*(SD)	730	1.83 (1.27)	236	1.90 (1.26)	0.755
*Parents*					
Sex, % Female, (*n)*	149	52.3 (78)	58	58.6 (34)	
Age (years), *M(SD)*^a^	149	49.2 (5.73)	58	50.1 (5.30)	
Highest level of education, % (*n*)	149		58		
No diploma		0.7 (1)		1.7 (1)	
Lower vocational		6.7 (10)		17.2 (10)	
Intermediate vocational		25.5 (38)		24.1 (14)	
Higher vocational or scientific (university)		67.1 (100)		56.9 (33)	
PBI	149		58		
Care *M*(SD)		31.37 (4.02)		29.47 (4.28)	0.002
Overprotection *M*(SD)		3.93 (2.49)		5.24 (2.89)	0.003
Lack of autonomy *M*)SD)		3.92 (2.50)		4.91 (2.50)	0.011
*Daily level*					
Maternal warmth *M*(SD)	948	5.70 (0.94)	406	5.73 (1.05)	0.687
Maternal criticism *M*(SD)	948	2.44 (1.43)	406	2.39 (1.34)	0.736
Paternal warmth *M*(SD)	785	5.38 (0.98)	252	5.40 (0.97)	0.632
Paternal criticism *M*(SD)	785	2.46 (1.40)	252	2.55 (1.46)	0.890

^a^Differences were tested by using appropriate non-parametric tests (retrospective level) or multilevel models (daily level)

PHQ-9 = Patient Health Questionnaire-9; CTQ = Childhood Trauma Questionnaire; PBI = Parental Bonding Inventory

### EMA

All participants received four questionnaires a day for 14 consecutive days (56 in total) on the Ethica app on their own smartphone. Questionnaires were triggered between 7AM and 9.30PM on weekdays and 9AM and 9.30PM on weekend days according to a standardized trigger schedule (detailed information in Appendix 3 and full codebook of EMA: https://osf.io/dcemq/). The EMA of RE-PAIR was conducted between September 2018 and March 2022. As compensation for EMA, parents received €20,- and HCs received €10,-. DEP adolescents did not receive compensation for the EMA since the assessments were considered as valuable information that could generate new insights and potentially facilitate the treatment due to possible self-insight. Treatment was not part of RE-PAIR and took place in regular clinical outpatient settings. To limit potential confounding influence of treatment, families who received parenting interventions or family-based treatment were not able to participate in RE-PAIR. Additionally, six gift vouchers of €75,- were raffled among all participating families based on compliance of EMA questionnaires completed by the family.

### Compliance

DEP adolescents fully completed 1193 (63.8%) of the delivered questionnaires and answered on average 35.1 questionnaires (*SD* = 13.9, range 11–55). In 554 cases (46.4% of answered questionnaires), adolescents indicated that they had interacted with one or both parents who participated in the EMA of RE-PAIR (*M* = 16.3 parent-adolescent interactions per participant, Range = 2–33). Parents fully completed 2329 (72.8%) of the delivered questionnaires and answered on average 40.2 questionnaires (*SD* = 9.7, range 11–53).

HCs fully completed 2930 (68.3%) of the delivered questionnaires and answered on average 37.1 questionnaires (*SD* = 11.2, range 3–55). In 1426 cases (48.7% of answered questionnaires), adolescents indicated that they had interacted with one or both parents who participated in the EMA of RE-PAIR (*M* = 18.1 parent-adolescent interactions per participant, Range = 3–42). This did not differ significantly from DEP adolescents (*p* = .334). Parents fully completed 6582 (80.5%) of the delivered questionnaires and answered on average 44.2 questionnaires (*SD* = 8.2, range 16–56). No participants were excluded based on missing data and all completed EMA data was retained for analyses.

## Measures

*Momentary positive and negative affect*. Adolescents rated their momentary affect using an adapted and shortened four-item version of the Positive and Negative Affect Schedule for Children [PANAS-C; 33, 34]. Two positive affect states (happy and relaxed) and two negative affect states (sad and irritated) were assessed by asking “How do you feel at this moment?” followed by: “Happy”, “Relaxed”, “Sad”, and “Irritated”. Answers were given on a 7-point Likert type scale ranging from 1 (*not at all*) to 7 (*very*). An average score of the two positive affect states was calculated, with the two items being strongly correlated at the between person-level, *r* = .830, *p* < .001, and moderately at the within-person level, *r* = .463, *p* < .001. An average score of the two negative affect states was calculated, with the two items being strongly correlated at the between person-level, *r* = .765, *p* < .001, and moderately at the within-person level, *r* = .335, *p* < .001. See Appendix 4 for correlations.

*Momentary positive and negative affect during parent-adolescent interaction*. Adolescents were asked with whom they spoke last to or with since the last beep and could select parents, friends, others, or no one. If they indicated they spoke last to or with mother, father or both follow-up questions were asked about this interaction. Adolescents rated their momentary affect during the interaction with an adapted and shortened five-item version of the Positive and Negative Affect Schedule for Children [PANAS-C; 33, 34]. Two positive affect states (happy and relaxed) and three negative affect states (sad, irritated, and guilty) were assessed by asking “How did you feel during this contact?” followed by: “Happy”, “Relaxed”, “Sad”, “Irritated”, and “Guilty”. Guilt, often part of or accompanying adolescent depression [35], was only assessed after interactions since parents and parenting can induce guilt during interactions [5]. Answers were given on a 7-point Likert type scale ranging from 1 (*not at all*) to 7 (*very*). For the current study, only answers about interactions with parents who participated in the EMA were included. An average score of the two positive affect states was calculated, with the two items being strongly correlated at the between person-level, *r* = .838, *p* < .001, and moderately at the within-person level, *r* = .428, *p* < .001. An average score of the three negative affect states was calculated, with the three items being strongly correlated at the between person-level, range *r* = .543–0.670, *p* < .001, and moderately to low at the within-person level, range *r* = .161–0.335, *p* < .001. See Appendix 4 for correlations.

*Parenting during parent-adolescent interaction.* Adolescents rated parenting behavior during the interaction, if they indicated that they spoke last to or with their parent(s), by answering the questions “How well did your mother/father listen to you?”, “How well did your mother/father understand you?”, “How critical was your mother/father towards you?”, and “How dominant was your mother/father?”. Answers were given on a 7-point Likert type scale with answer categories ranging from 1 (*not at all*) to 7 (*very*).

Similarly, if parents indicated that they spoke last to or with their adolescent since the last beep, they rated their own parenting behavior during the interaction. They answered the questions “How well did you listen to your child”, “How well did you understand your child?”, “How critical were you towards your child?”, and “How dominant were you towards your child?”. Answers were given on a 7-point Likert type scale with answer categories ranging from 1 (*not at all*) to 7 (*very*). Two subscales were created for parents and adolescents separately, parental warmth and parental criticism. An average score of listening and understanding behavior during the interaction was calculated to assess parental warmth, with the two items being strongly correlated at the between person level, range *r* = .763–0.949, *p* < .001 and moderately to strongly at the within-person level, range *r* = .415–0.697, *p* < .001. An average score of critical and dominant behavior during the interaction was calculated to assess parental criticism, with the two items being strongly correlated at the between person level, range *r* = .520–0.724, *p* < .001 and strongly at the within-person level, range *r* = .562–0.657, *p* < .001. See Appendix 4 for correlations.

*Daily parenting*. In the last questionnaire of each day, adolescents indicated whether they spoke to a parent during that day and with whom (i.e., mother, father, stepmother, stepfather). Adolescents rated daily parenting for each parent they spoke to by answering the questions: “Throughout the day, how critical was your mother/father towards you?” and “Throughout the day, how warm/loving was your mother/father towards you?” Answers were given on a seven-point Likert type scale ranging from 1 (*not at all*) to 7 (*very*). Similarly, parents indicated whether they spoke to the participating adolescent in the last questionnaire of each day. Parents rated their own behavior by answering the questions “Throughout the day, how critical were you towards your child?” and “Throughout the day, how warm/loving were you towards your child?” Answers were given on a seven-point Likert type scale with answer categories ranging from 1 (*not at all*) to 7 (*very*).

*Depressive symptoms*. The Patient Health Questionnaire [PHQ-9; 36] was used to assess adolescent depressive symptoms the past two weeks as part of the online questionnaires adolescents had to complete before the research day in the lab. The items are based on nine DSM-IV criteria for depression and are rated as 0 (*not at all)* to 3 (*nearly every day*). One item (item 8; moving or speaking slowly or being so fidgety or restless) was split in two items and the maximum score of these two items was included. Sum scores range from 0 to 27 and a score above 10 is suggestive of the presence of depression [37]. Cronbach alpha was 0.94.

*Parental bonding.* To assess the parent-adolescent bond, adolescents completed the Dutch version of the Parental Bonding Inventory [PBI; 38] as part of the online questionnaires. Adolescents reported on the bond with mothers and fathers separately and only reports about parents who participated in the EMA were included. The instrument consisted of 25 items and included three subscales: care (12 items), overprotection (6 items), and lack of autonomy (7 items) [39]. Answers were given on a 4-point Likert scale ranging from 0 (*very like*) to 3 (*very unlike*). Since wording of one item of lack of autonomy subscale deviated from the original PBI and showed inconsistent loading [39], we excluded the item from the subscale in the current study. After reverse coding 13 items, a sum score per subscale was calculated and higher scores indicated more care, overprotection, and lack of autonomy. Cronbach’s alphas in the current sample regarding care, overprotection, and lack of autonomy with mothers were α = 0.88, α = 0.62, and α = 0.82 respectively. Regarding care, overprotection, and lack of autonomy with fathers Cronbach’s alphas were α = 0.87, α = 0.59, and α = 0.71 respectively.

In order to also assess the parent-adolescent bond from the perspective of parents we rephrased the items of the PBI. Cronbach’s alphas for PBI of parents were α = 0.77 for care, α = 0.58 for overprotection, and α = 0.64 for lack of autonomy.

*Childhood emotional maltreatment.* To assess experienced childhood emotional maltreatment, adolescents filled out the Dutch version of the Childhood Trauma Questionnaire short form as part of the online questionnaires [CTQ-SF; 40, 41]. The full CTQ-SF consists of 25 items including five subscales. The current study uses two subscales: emotional abuse and emotional neglect. Both subscales consist of five items and were answered on a Likert scale, ranging from 1 (*never true*) to 5 (*very often true*). Five items were reverse coded before calculating a sum score per subscale. Higher scores indicating more experienced childhood emotional maltreatment. Overall, the CTQ-SF has been shown to have high reliability and validity [41, 42]. Validity in an adolescent sample has also been shown [43]. In the current sample Cronbach’s alphas were α = 0.82 for emotional abuse and α = 0.84 for emotional neglect.

### Preregistered analyses

Our analysis plan, including power analyses, was preregistered online (https://osf.io/qjyp5). As the amount of observations of interactions of adolescents with fathers was less than expected, we performed some sensitivity checks (see Appendix 5). For the analyses we used R version 4.0.1 [44] and for the multilevel package version 2.6 with ML estimation. Level 1 predictors were person-mean centered, following guidelines proposed by [45] and [46].

To account for the nestedness of the data (i.e., measurements nested in individuals) we used multiple multilevel models. We ran two-level models instead of three-level models (moments nested in days nested in individuals) as some adolescents on some days did not or once indicated an interaction with their parent(s). To examine whether adolescent momentary positive and negative affect (in general and during parent-adolescent interactions) and momentary parental warmth and criticism during parent-adolescent interactions differed between families with a DEP adolescent and HCs (aim 1) we tested eight models including adolescents’ reports and four including parents’ reports. Although not preregistered, we also compared momentary, daily, and retrospective reports of parenting between the two groups to explore recall bias using multilevel models (momentary and daily level) and appropriate non-parametric tests (retrospective reports). To investigate the within-person association between perceived parenting behavior and adolescent affect during parent-adolescent interactions (aim 2), we added the person-mean centered scores of perceived maternal warmth, perceived maternal criticism, perceived paternal warmth, and perceived paternal criticism to the unconditional random intercept models of positive and negative affect separate (eight models). Next, in each model, variation was allowed around the slope to examine heterogeneity. Likelihood ratio tests were used to assess differences in fit of the models [following guidelines of 47]. To assess whether the association between parenting and adolescent affect during parent-adolescent interactions was stronger for DEP adolescents (aim 3), we added the binary variable clinical status (0 = HCs, 1 = DEP adolescents) to the model as main effect and in interaction with perceived parenting. Lastly, we explored whether the association between parenting and adolescent affect during parent-adolescent interactions was stronger for adolescents with more depressive symptoms. This level 2 predictor was grand-mean centered.

Correlation structure corCAR1 was added in all models to take into account unequally spaced time intervals [48].

## Results

### Differences between DEPs and HCs

**Fig. 1:**
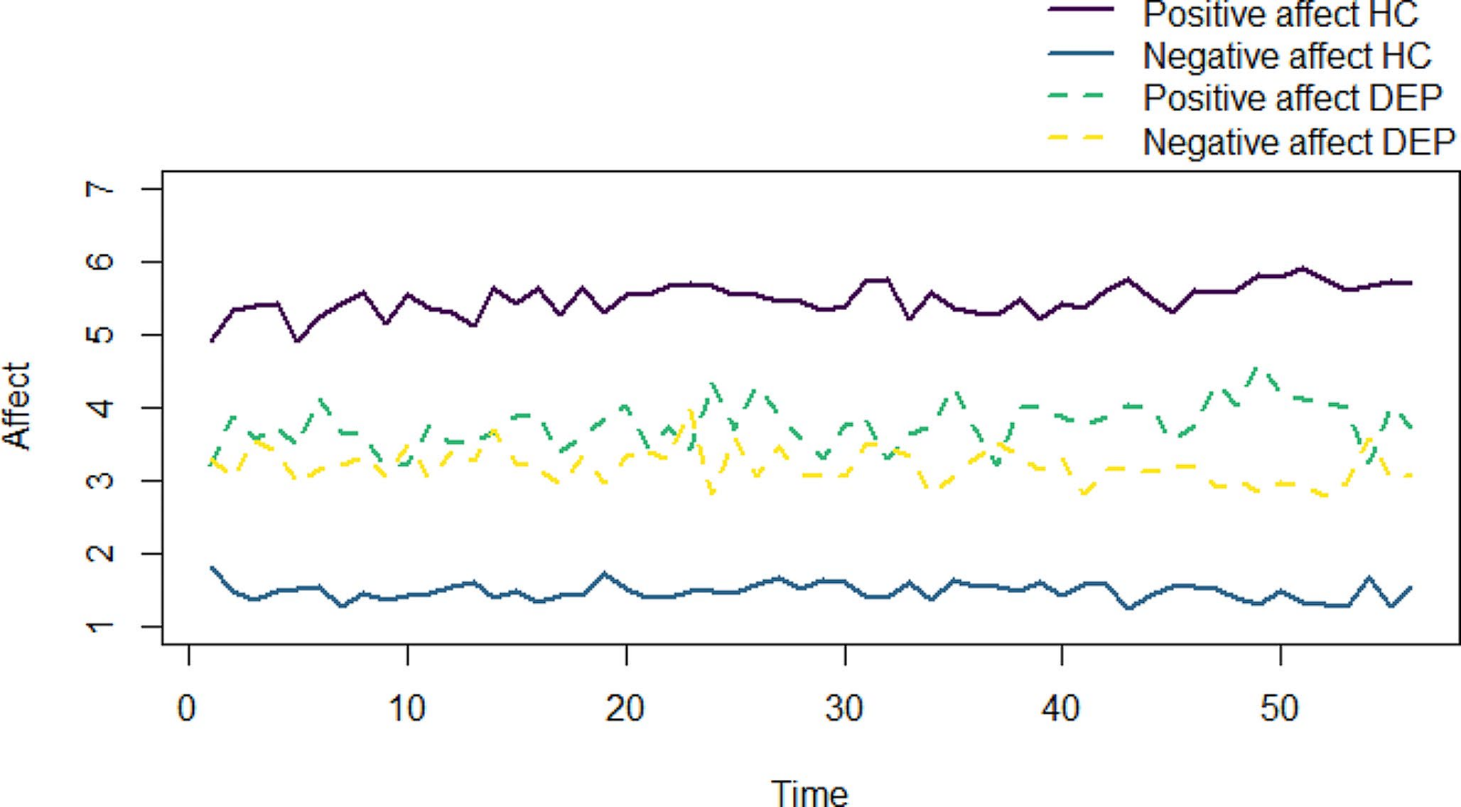
Average fluctuations of momentary positive and negative affect of adolescents over time per group, HC adolescents and DEP adolescents

**Fig. 2:**
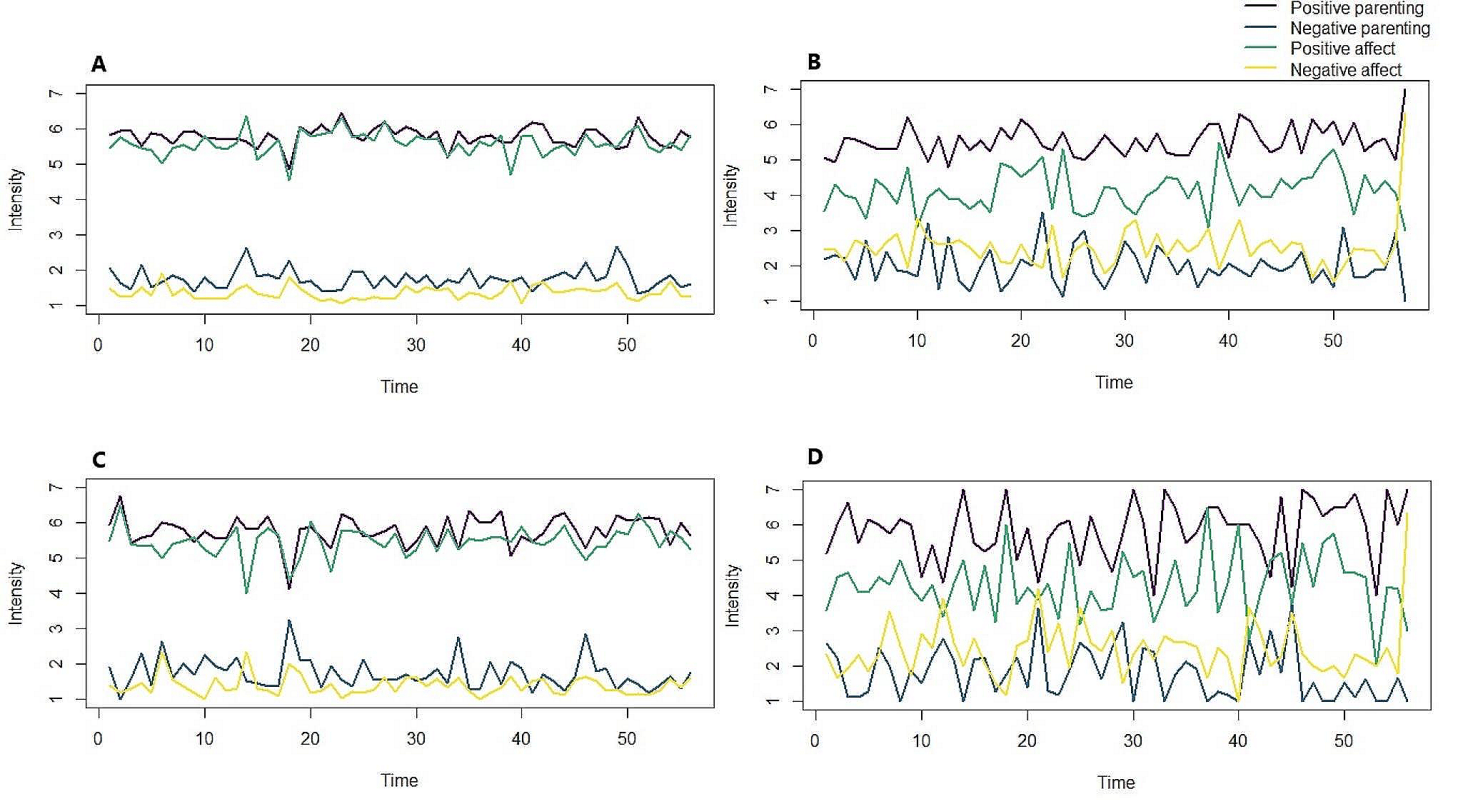
Average fluctuations of momentary adolescent affect and perceived parenting during parent-adolescent interactions per group over time (observations). Panel **A** and **B** represent interactions with mothers reported by HC adolescents and DEP adolescents respectively. Panel **C** and **D** represent interactions with fathers reported by HC adolescents and DEP adolescents respectively

Descriptive statistics of the study variables and results of multilevel models are presented in Table 2 (see Appendix 6 for correlations). DEP adolescents reported significantly less momentary positive and more negative affect than HCs (*p’*s < 0.001, see Fig. 1). During parent-adolescent interactions, DEP adolescents reported significantly less positive and more negative affect than HCs (*p*’s < 0.001, see Fig. 2). DEP adolescents did not differ from HCs in their perceptions of perceived parental warmth and parental criticism of mothers and fathers during parent-adolescent interactions (all *p*’s > 0.050, see Fig. 2). Similarly, mothers’ and fathers’ own perception of parental warmth and criticism did not differ between parents of DEP adolescents and HCs (all *p*’s > 0.050).

**Table 2:** Descriptive statistics of study variables and results of multilevel models to test differences between groups

	HC		DEP		Difference
	*Obs*	*M*(SD)	*Obs*	*M*(SD)	*Estimate* (*p)*
*Adolescents*					
Positive affect	2947	5.47 (1.13)	1212	3.77 (1.53)	-1.694 (< 0.001)
Negative affect	2946	1.47 (0.91)	1210	3.20 (1.54)	1.710 (< 0.001)
Positive affect during parent-adolescent interaction	1425	5.57 (1.15)	552	4.09 (1.40)	-1.483 (< 0.001)
Negative affect during parent-adolescent interaction	1425	1.33 (0.67)	551	2.50 (1.19)	1.183 (< 0.001)
Maternal warmth during parent-adolescent interaction	1053	5.79 (1.19)	438	5.47 (1.23)	-0.348 (0.062)
Maternal criticism during parent-adolescent interaction	1053	1.74 (1.14)	438	2.01 (1.35)	0.289 (0.118)
Paternal warmth during parent-adolescent interaction	624	5.79 (1.21)	194	5.72 (1.36)	-0.160 (0.496)
Paternal criticism during parent-adolescent interaction	624	1.69 (1.09)	194	1.86 (1.29)	0.194 (0.350)
*Parents*					
Maternal warmth during parent-adolescent interaction	798	5.75 (1.03)	446	5.64 (1.15)	0.002 (0.991)
Maternal criticism during parent-adolescent interaction	798	2.10 (1.40)	445	2.21 (1.28)	0.081 (0.658)
Paternal warmth during parent-adolescent interaction	449	5.65 (0.91)	163	5.26 (1.17)	-0.193 (0.267)
Paternal criticism during parent-adolescent interaction	449	2.29 (1.30)	163	2.36 (1.35)	-0.002 (0.993)

*Note* Healthy controls (*n* = 79) and their parents (*n* = 149), adolescents with depression (*n* = 34) and their parents (*n* = 58)

On the daily level, DEP adolescents reported lower levels of perceived parental warmth of mothers and fathers than HCs (*p*’s < 0.05, see Table 1), whereas levels of perceived criticism did not significantly differ. Parents’ self-reported daily parental warmth and criticism (assessed at the end of the day) did not significantly differ between the two groups. Retrospectively, using appropriate non-parametric tests, DEP adolescents reported higher levels of parental emotional abuse and neglect during their childhood compared to HCs (*p*’s < 0.001) and less care and more overprotection from mothers and fathers than HCs (*p’*s < 0.01). Parents of DEP adolescents also reported less care, more overprotection, and more lack of autonomy compared to parents of HCs (*p*’s < 0.05).

### Within-person associations

As indicated by the intraclass correlations (ICC) 57.4% of the variance in adolescent negative affect and 60.8% of the variance in adolescent positive affect was due to differences between adolescents, and 42.6% and 39.2% due to within-person fluctuations over time. Examination of the within-person association between perceived parenting behavior and affect during momentary parent-adolescent interactions (aim 2) showed that when adolescents perceived their mothers and fathers to show more warmth or less criticism during interactions, they also reported more positive and less negative affect (all *p*’s < 0.001, see Appendix 7). Next, we allowed variation around the slope of perceived parenting in each model and likelihood ratio tests indicated that this improved the model fits significantly (all *p*’s < 0.001), This indicates that adolescents differed substantially in the extent to which perceived parental warmth and criticism of mothers and fathers were associated with positive and negative affect.

**Fig. 3:**
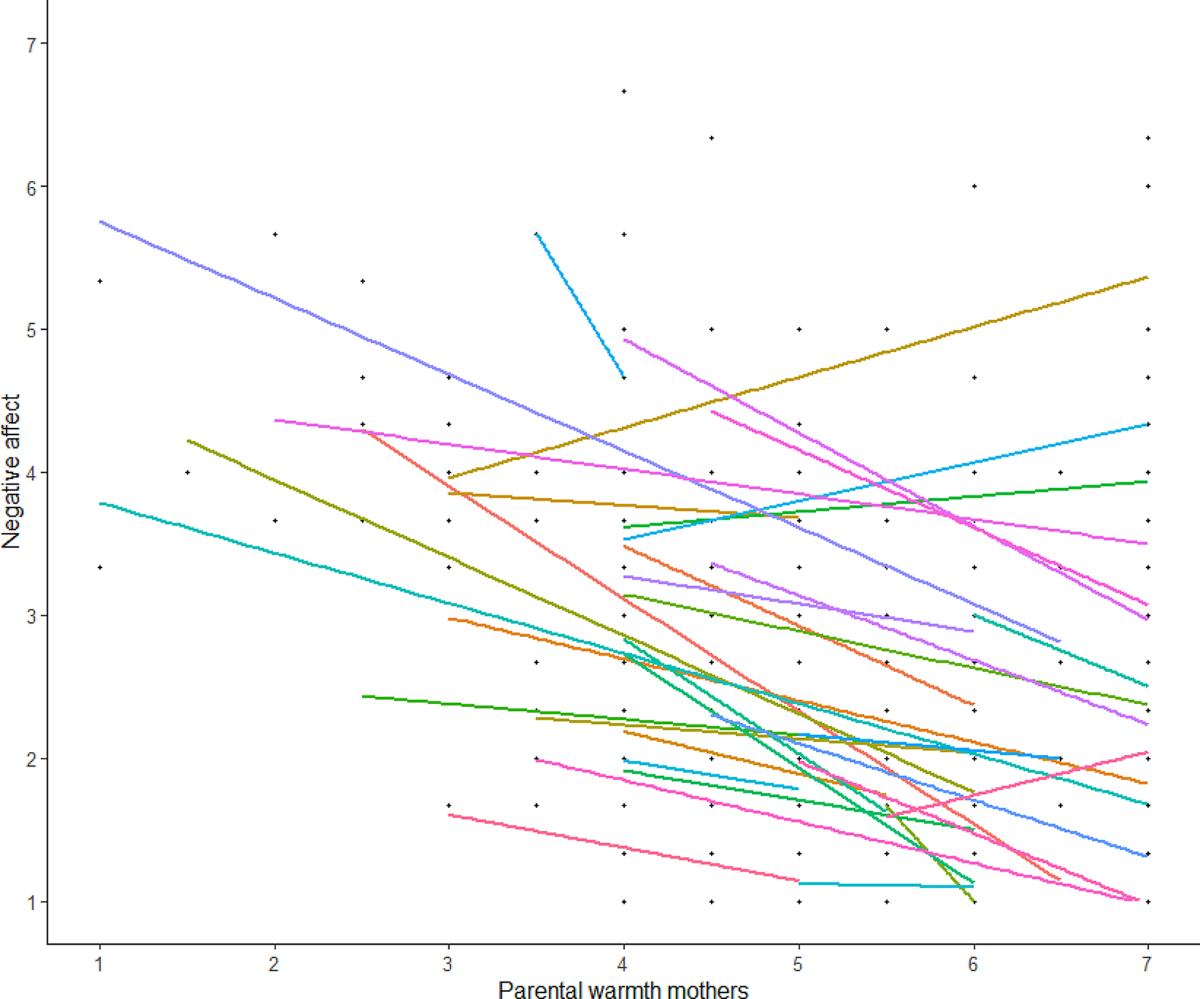
Individual-level associations between parental warmth of mothers and negative affect during momentary parent-adolescent interactions for DEP adolescents. Each line represents one person

To examine whether the association between perceived parenting and adolescent affect during momentary parent-adolescent interactions differed between DEP adolescents and HCs (aim 3), we added clinical status (being diagnosed with depression or not) to the models as well as an interaction of clinical status with perceived parenting. Results are displayed in Table 3. In all models, there was no significant interaction between perceived parenting and clinical status, indicating that the link between perceived parenting and adolescent affect did not differ between DEP adolescents and HCs (see Appendix 8 for figures). Further inspection of these associations in DEP adolescents indicated that even within this group, there are individual differences in how parenting and adolescent affect are related. This is illustrated in Fig. 3 in which individual associations between parental warmth of mothers and negative affect during momentary parent-adolescent interactions were plotted for DEP adolescents.

**Table 3:** Parental warmth and criticism and adolescent positive and negative affect during parent-adolescent interactions and the moderating role of depression

	Maternal warmth	Paternal warmth	Maternal criticism	Paternal criticism
*Adolescent positive affect*				
*Fixed effects: estimate (SE)*				
Intercept	5.457*** (0.102)	5.463*** (0.120)	5.460 *** (0.102)	5.465*** (0.120)
Perceived parenting	0.441*** (0.049)	0.447*** (0.061)	−0.280*** (0.049)	−0.351*** (0.069)
Clinical status	−1.436*** (0.186)	−1.472*** (0.238)	−1.434*** (0.186)	−1.472*** (0.238)
Perceived parenting*clinical status	−0.072 (0.087)	0.008 (0.121)	0.037 (0.085)	0.045 (0.140)
*Random effects*				
Between-person variance	0.726	0.866	0.714	0.856
Within-person variance	0.644	0.495	0.724	0.559
Random effect variance	0.076	0.103	0.065	0.118
*Adolescent negative affect*				
*Fixed effects: estimate (SE)*				
Intercept	1.413*** (0.077)	1.425*** (0.080)	1.414*** (0.077)	1.426*** (0.080)
Perceived parenting	−0.225*** (0.035)	−0.183*** (0.046)	0.257*** (0.036)	0.239*** (0.042)
Clinical status	1.155*** (0.140)	1.165*** (0.158)	1.154*** (0.141)	1.166*** (0.159)
Perceived parenting*clinical status	−0.076 (0.061)	−0.027 (0.091)	0.033 (0.064)	0.151 (0.084)
*Random effects*				
Between-person variance	0.407	0.366	0.418	0.378
Within-person variance	0.359	0.288	0.333	0.276
Random effect variance	0.036	0.059	0.043	0.035

*Note* Models concerning adolescent-mother interactions *N* individuals = 112, *N* observations = 1491. Models concerning adolescent-father interactions *N* individuals = 90, *N* observations = 818

*p < .05. **p < .01. ***p < .001

We furthermore explored whether the association between parenting and adolescent affect during parent-adolescent interactions differed based on severity of depressive symptoms instead of clinical status. Findings were similar compared to clinical status and indicated that the link between perceived parenting and adolescent affect during parent-adolescent interactions did not differ between adolescents based on the severity of depressive symptoms. Full model results are presented in Appendix 9.

### Sensitivity analyses

In addition to our preregistered analyses, we conducted three post hoc sensitivity analyses. First, to tease apart within- from between-person associations, we calculated the person-mean of perceived parenting and added the grand-mean centered score to the model including perceived parenting and clinical status. Average perceived parenting was significantly associated with adolescent affect (all *p*’s < 0.01) in all models. Results on daily perceived parenting and clinical status did not change from previous models (see Appendix 10). Second, to control for the possible influence of time, we added the observation variable (a count variable ranging from 1 to 56) to the models including perceived parenting and clinical status (see Appendix 11). Results on daily perceived parenting and clinical status remained the same, but time was a significantly related with adolescent positive affect (*p*‘s < 0.01) except in the model with parental criticism of fathers. Third, to elucidate whether the association between perceived parenting and adolescent affect during parent-adolescent interactions differed between boys and girls, we included perceived parenting, clinical status (as main effect), sex, and an interaction between sex and perceived parenting in the models. The interaction between sex and perceived parenting was not significant, indicating that the link between perceived parenting and adolescent affect did not differ between boys and girls (see Appendix 12 for full model results). Sex itself was also not significantly related to adolescent positive and negative affect in the models including maternal warmth and criticism. However, when inspecting the models focusing on the interactions between adolescents and fathers, adolescent girls reported less positive and more negative affect than boys during interactions with their fathers (all *p*’s < 0.050).

## Discussion

We examined the moment-to-moment experiences of adolescent affect and parenting during parent-adolescent interactions in a clinical sample of families with DEP adolescents in comparison to families with HCs. In line with our preregistered hypotheses and some previous research [22, 23], we found that DEP adolescents experienced lower levels of positive affect and higher levels of negative affect than HCs throughout the days as well as during parent-adolescent interactions. As illustrated in Fig. 1, on average DEP adolescents reported little below the middle of the scale (ranging from not at all to very) which may indicate a more flat or blunted affect. This seems to be partly in line with the Emotion Context Sensitivity theory that proposes that depression flattens emotions in general [49].

In contrast to our hypotheses, momentary levels of reported parental warmth and criticism during parent-adolescent interactions did not differ between the two groups, not from the perspective of the adolescent nor from the parent (i.e., mother and father). On a daily level, parental criticism did not differ between the two groups, but DEP adolescents *did* perceive their parents to be less warm. Moreover, adolescents with DEP also perceived their relationship with mothers and fathers as more negative (e.g., less care, more overprotection, more emotional abuse and neglect) as indicated on the retrospective questionnaires compared to HCs. Parents of DEP adolescents themselves also reported less parental care and more overprotection on the retrospective questionnaires than parents of HCs. These discrepancies are intriguing, with the retrospective reports being in line with previous findings based on retrospective questionnaires (and observations in the lab) that also indicate that parent-adolescent interactions in families with DEP adolescents are less supportive and more conflictual [e.g., 17, 50] and lower in parental care [15, 51]. Thus, findings on retrospective questionnaires do not necessarily match momentary assessments [18]. When adolescents are asked to report retrospectively on parenting, their memories may be negatively biased by their mood [9]. Parents of DEP adolescents have shown to be more worried about their child and question their own parenting abilities [52], which may explain differences in momentary versus retrospective parenting report of parents. Assessing parenting at the momentary level with EMA may reduce these biases.

Our findings furthermore indicate the important association between parenting and adolescent’s well-being, also for DEP adolescents. When adolescents perceived their parents as more warm or less critical during interactions they also reported more positive and less negative affect, or vice versa, supporting previous findings in community samples at the momentary [31] or daily level [29, 30]. The momentary associations between parenting and affect in the current study did not differ between HCs and DEP adolescents. A recent study on parenting and affect during momentary parent-adolescent interactions, based on a community sample, reported similar results [31]. The abovementioned biases may play a role here as daily reports of parenting still involve some recollection, including the inherent biases, while these do not apply to momentary assessments.

Another important finding is that we found substantial variation between adolescents, indicating that the strength or direction of how warm or critical parenting is associated to adolescent affect differs between adolescents. Even within our sample of DEP adolescents, this heterogeneity was observed, showing that the association between parental warmth and criticism differed between DEP adolescents. This aligns with a previous finding also showing different patterns in adolescents who report clinically relevant depressive symptoms [29]. Studies using more person-centered and idiographic approaches are needed [53] to better understand these patterns and translate them into implications for clinical practice.

A unique feature of the current study was that we assessed parental warmth and criticism of mothers and fathers separately. Despite family system theories proposing adolescent-mother and adolescent-father dyads being distinct subsystems [6, 54] and suggestions that parenting roles of mothers and fathers may differ [e.g., 55], not many studies have assessed parenting of both mothers and fathers. Our results suggest that perceived parental warmth and criticism of mothers *and* fathers are important for adolescent well-being. Interestingly, sensitivity analyses (in the supplementary materials) indicated that girls reported more negative and less positive affect in interactions with fathers than boys. These findings highlight the need to assess family dynamics of mothers, fathers, and adolescents together, as well as taking into account sex of adolescents.

As our study provides first insights into the momentary experiences of families with DEP adolescents by monitoring parent-adolescent interactions and includes not only adolescents’ perceptions of parenting of mothers and fathers separately but also parents’ own perception, findings are relevant for clinical practice. Since DEP adolescents do seem to benefit from parental warmth in daily life, interventions on adolescent depression may benefit from the involvement of parents, both mothers and fathers. A recent meta-analysis has shown that the involvement of parents in treatment of adolescent depression can increase the efficacy of individual CBT [56]. These family interventions could include psychoeducation to inform parents about how depression and how cognitive biases can influence adolescents’ experiences of daily life, and foster a warm family climate, limiting parental rejection, and criticism. Moreover, given the substantial variation in how parenting and adolescent affect is related and previous findings that perceptions of adolescents and parents differ [57, 58], exploring the needs of the adolescent in treatment and discussing them with parents also seems an important ingredient. This could yield more understanding of each other’s perception and behavior as well as aligning what adolescents need or want and what parents can provide.

Some limitations should be acknowledged that may provide directions for future studies. Our sample was fairly homogenous, with the majority of adolescents and parents being born in the Netherlands. Furthermore, our sample of families with a DEP adolescent might be biased. Families who decided to participate in the study, focusing on parent-adolescent interactions, may not be families with harsh or neglecting parenting behavior, thereby resulting in an underestimation of negative parent-adolescent interactions among families with DEP adolescents. Also, even though common comorbid disorders, such as anxiety disorders, were allowed, we excluded DEP adolescents with forms of comorbid psychopathology, such as autism, that may directly influence parent-adolescent interactions. As a result, findings may deviate from naturalistic patterns in adolescents which limits interpretation and generalizability. Although future studies may strive to include a more diverse, representative sample of DEP adolescents, it is also important to mention that the inclusion of comorbid disorders may pose challenges when it comes to elucidating the specific associations with adolescent depression and parent-adolescent interactions. Moreover, although we were able to assess experiences of parent-adolescent interactions in their natural context by using EMA, it may also have resulted in collecting data of interactions about mundane matters (e.g., who is unloading the dishwasher) that may not have a large impact on adolescents’ affect. Future studies may benefit from gaining additional information about the *content* of the interactions. Lastly, due to limited power we have to be cautious in interpreting the findings concerning interactions with fathers. We were also not able to test the direction of effects, but focused on concurrent associations during momentary parent-adolescent interactions. Future work assessing the direction of effects could result in more specific implications for clinical practice.

## Conclusion

Parenting has been consistently associated with adolescent depression, but most research to date has used retrospective questionnaires concerning macro-time intervals. With the use of EMA and inclusion of families with a DEP adolescent, we showed that DEP adolescents overall reported more negative and less positive affect than HCs. Generally, perceived parental warmth and criticism and affect during parent-adolescent interactions co-fluctuated. This association did not differ between DEP adolescents and HCs, even though DEP adolescents and their parents did indicate more negative parenting (e.g., less care and more overprotection) in the retrospective questionnaires. These findings indicate that adolescents generally do seem to benefit from parental warmth, while the discrepant findings also support the idea that a negativity bias may have affected the retrospective reports of parenting. Clinicians should facilitate the communication of needs and perspectives between adolescents and parents. The study further supports the idea that the extent to which parenting processes relate to adolescent affect differs per family and therefore calls for a more person-centered and idiographic approach in research to guide family interventions.

## Electronic supplementary material

Below is the link to the electronic supplementary material.

Supplementary Material 1

## Data Availability

The de-identified data, analysis scripts, and materials for this study will be made available on DataverseNL.
