# Supplementary material for: A closer look into the affect dynamics of adolescents with depression and the interactions with their parents: An ecological momentary assessment study
Source: Eur Child Adolesc Psychiatry. 2024 May 18;33(12):4259–72. doi: 10.1007/s00787-024-02447-1 (PMC11618135; doi:10.1007/s00787-024-02447-1)
Supplement: Supplementary file 1 — Supplementary Material 1 [file 787_2024_2447_MOESM1_ESM.docx]

**Supporting information**

**A closer look into the affect dynamics of adolescents with depression and the interactions with their parents: An ecological momentary assessment study**

Loes H. C. Janssen^1,2^, Bart Verkuil^1,2^, Lisanne A.E.M. van Houtum^1,2^, Mirjam C.M. Wever^1,2^, Wilma, G. M. Wentholt^1,2^, & Bernet M. Elzinga^1,2^

*^1^Department of Clinical Psychology, Leiden University, Leiden, the Netherlands;*

*^2^Leiden Institute for Brain and Cognition (LIBC), Leiden, the Netherlands*

Contact corresponding author [l.h.c.janssen@fsw.leidenuniv.nl](mailto:l.h.c.janssen@fsw.leidenuniv.nl)

**Appendix 1**

Detailed information on recruitment and study procedure

HC families were recruited via networks of employees of Leiden University, flyers at public places, and advertisements in (online) media. Families interested in participating could contact the RE-PAIR research team via the website, telephone, or mail. Information letters were sent to the families and subsequently researchers called parents and adolescents to provide more information and administer screening questions. If all inclusion and no exclusion criteria were met, an appointment was scheduled for a research day in Leiden.

Families with an adolescent with a depression were recruited via several mental health organizations and (social) media. If the family situation and symptoms of the adolescent seemed to fit the study criteria, the RE-PAIR study was introduced to the family. When the family was interested in participation, an appointment was made with the adolescent to administer the K-SADS-PL. If the adolescent met the criteria, a phone call was made with the parents to make an appointment for the research day in Leiden.

Adoptive, foster, and stepparents (*n* = 16) were allowed to participate if they were involved in the upbringing of the adolescent for at least five years and if adolescents perceived the parent as a primary caregiver. For reasons of clarity, they will be referred to as mothers and fathers.

During the research day, adolescents and parents received face-to-face instructions about the EMA procedure and researchers assisted in installing the Ethica Data application. Each family member also received written instructions and their individual account information. Generally, the EMA started the next Monday after the research day, but three families with an adolescent with a depression started on a different day due to a technical error or medical event. For healthy controls and their parents, EMA was postponed in case of holidays and exam weeks of the adolescents to the first Monday thereafter. For adolescents with a depression and their parents, EMA was only postponed in case of going on a holiday (i.e., not being at home) because delays in the EMA period could result in an overlap with start of treatment or improvements during treatment, interfering with the assessments.

‌

**Appendix 2**

Detailed information on comorbidity of adolescents and psychopathology of parents.

Of the DEP adolescents, 28 (82.4%) also had at least one current comorbid disorder, such as an anxiety disorder (*n* = 19; specific phobia, social anxiety disorder, panic disorder, agoraphobia, or generalized anxiety disorder) and 18 adolescents (52.9%) had two or more current comorbid disorders.

Parents were screened on current and past psychopathology with the Mini International Neuropsychiatric Interview [M.I.N.I.; 1] during the research day in the lab by trained students. 35 of all 207 parents (16.9%) fulfilled criteria for at least one current mental disorder (*n* = 17 (11.4%) parents of HCs; *n* = 18 (31.0%) parents of DEP adolescents) of which 13 fulfilled criteria for two or more disorders. Of these 35 parents, 6 parents (*n* = 4 parents of DEP adolescents) fulfilled criteria for current MDD and 5 for dysthymia (*n* = 4 parents of DEP adolescents).

Beside MDD or dysthymia, other current diagnoses parents’ met criteria for were: mania, panic disorder, agoraphobia, social phobia, obsessive compulsive disorder, posttraumatic stress disorder, alcohol/drug abuse/misuse, psychotic disorder, and generalized anxiety disorder.

Of all parents, 75 (36.2%) fulfilled criteria for at least one past mental disorder (*n* = 44 parents of HCs; *n* = 31 parents of DEP adolescents) and 30 fulfilled criteria for two or more disorders. Of these 75 parents, 47 parents (*n* = 18 parents of DEP adolescents) fulfilled criteria for past depressive disorder and 3 for dysthymia (*n* = 2 parents of DEP adolescents).

**References**

[1] Sheehan DV, Lecrubier Y, Sheehan KH, Amorim P, Janavs J, Weiller E, Hergueta T, Baker R, Dunbar GC. The Mini-International Neuropsychiatric Interview (M.I.N.I.): the development and validation of a structured diagnostic psychiatric interview for DSM-IV and ICD-10. J Clin Psychiatry. 1998;59 Suppl 20:22-33;quiz 34-57

**Appendix 3**

Detailed information about triggering schedule, expiration time, and monitoring processes

Participants filled out questionnaires on their smartphone using the Ethica app for 14 consecutive days between 7AM and 9.30PM on weekdays and 9AM and 9.30PM on weekend days according to a standardized trigger schedule. Participants received four questionnaires each day (56 in total), signaled by a notification, and were instructed to complete the questionnaires as quickly as possible. All questionnaires consisted of questions on their whereabouts, affect, and contact with others. The first questionnaire of each day was sent at 7AM on weekdays and 9AM during weekend days and expired after 120 min. The second and third questionnaires were sent at a random time point, with the second between 12 noon and 1PM, and the third between 4PM and 7PM. Both expired after 60 min. The last questionnaire of each day was sent to adolescents at a random time point between 8.15PM and 8.45PM and to parents between 9PM and 9.30PM, both expired after 180 min. The first questionnaire of each day additionally included questions about sleep and the last questionnaire of each day about self-image, parenting, and substance use (e.g., coffee, alcohol) throughout the day.

The questionnaires consisted of minimal 14 items, 13 closed and 1 open, and maximal 45 items, 44 closed and 1 open. Number of items depended on role (parent or adolescent), branching, and type of questionnaire (morning, day, or evening). On average, filling out the questionnaires took healthy controls 2.21 min per questionnaire (*SD* = 2.73) and their parents 2.66 min per questionnaire (*SD* = 2.48), while for adolescents with a depression this was 2.53 min (*SD* = 3.23) and their parents 3.18 min (*SD* = 4.08). Multilevel models indicated that healthy controls and their parents completed the questionnaires in less time than adolescents with a depression and their parents (both *p*’s < .01).

Researchers monitored the EMA by checking daily whether participants received and completed questionnaires and were available for questions or problems via WhatsApp, telephone, and mail. On day 4, 7, and 11 of the EMA an update was sent to each participant about the personal adherence (percentage of completed questionnaires) as motivation. On the last day of the EMA, a message was sent to thank participants and remind them of the scheduled phone call after the EMA to evaluate. Participants did not receive automatic reminders for the questionnaires.

**Appendix 4**

|  |  | 2 | 3 | 4 | 5 | 6 |
| --- | --- | --- | --- | --- | --- | --- |
|  |  |  |  |  |  |  |
| 1. Happy |  | .463*** | -.511*** | -.386*** | .837*** | -.539*** |
| 2. Relaxed | .830*** |  | -.324*** | -.371*** | .872*** | -.427*** |
| 3. Sad | -.778*** | -.723*** |  | .335*** | -.482*** | .770*** |
| 4. Irritated | -.674*** | -.694*** | .765*** |  | -.441*** | .859*** |
| 5. Positive affect | .955*** | .958*** | -.784*** | -.716*** |  | -.561*** |
| 6. Negative affect | -.782*** | -.755*** | .963*** | .909*** | -.804*** |  |

Correlations of adolescent momentary affect

*Note*: Between-person correlations are presented under the diagonal, within-person correlations are presented above the diagonal.

* *p* < .05; ** *p* < .01; *** *p* < .001.

|  |  | 2 | 3 | 4 | 5 | 6 | 7 |
| --- | --- | --- | --- | --- | --- | --- | --- |
|  |  |  |  |  |  |  |  |
| 1. Happy |  | .428*** | -.489*** | -.433*** | -.191*** | .839*** | -.540*** |
| 2. Relaxed | .838*** |  | -.316*** | -.403*** | -.232*** | .851*** | -.463*** |
| 3. Sad | -.677*** | -.605*** |  | .335*** | .242*** | -.475*** | .723*** |
| 4. Irritated | -.705*** | -.675*** | .670*** |  | .161*** | -.494*** | .794*** |
| 5. Guilty | -.525*** | -.520*** | .599*** | .543*** |  | -.251*** | .579*** |
| 6. Positive affect | .959*** | .958*** | -.669*** | -.720*** | -.545*** |  | -.593*** |
| 7. Negative affect | -.748*** | -.698*** | .929*** | .853*** | .772*** | -.755*** |  |

Correlations of adolescent momentary affect during parent-adolescent interactions

*Note*: Between-person correlations are presented under the diagonal, within-person correlations are presented above the diagonal.

* *p* < .05; ** *p* < .01; *** *p* < .001.

Correlations of perceived parenting during parent-adolescent interactions reported by adolescents

|  |  | 2 | 3 | 4 | 5 | 6 | 7 | 8 | 9 | 10 | 11 | 12 |
| --- | --- | --- | --- | --- | --- | --- | --- | --- | --- | --- | --- | --- |
|  |  |  |  |  |  |  |  |  |  |  |  |  |
| 1. Listening mother |  | .695*** | -.358*** | -.386*** | .916*** | -.418*** | .575*** | .437*** | -.198*** | -.304*** | .540*** | -.279*** |
| 2. Understanding mother | .949*** |  | -.391*** | -.416*** | .925*** | -.453*** | .476*** | .612*** | -.309*** | -.344*** | .584*** | -.364*** |
| 3. Critical mother | -.620*** | -.650*** |  | .578*** | -.407*** | .896*** | -.183*** | -.240*** | .674*** | .374*** | -.227*** | .586*** |
| 4. Dominant mother | -.536*** | -.531*** | .750*** |  | -.436*** | .881*** | -.206*** | -.278*** | .425*** | .641*** | -.260*** | .594*** |
| 5. Maternal warmth | .987*** | .988*** | -.644*** | -.540*** |  | -.474*** | .566*** | .567*** | -.273*** | -.349*** | .606*** | -.347*** |
| 6. Maternal criticism | -.615*** | -.628*** | .929*** | .942*** | -.630*** |  | -.216*** | -.288*** | .624*** | .557*** | -.271*** | .659*** |
| 7. Listening father | .726*** | .687*** | -.428*** | -.459*** | .715*** | -.473*** |  | .697*** | -.427*** | -.457*** | .918*** | -.492*** |
| 8. Understanding father | .761*** | .750*** | -.505*** | -.482*** | .765*** | -.526*** | .944*** |  | -.465*** | -.463*** | .924*** | -.516*** |
| 9. Critical father | -.373*** | -.411*** | .722*** | .571*** | -.397*** | .687*** | -.441*** | -.502*** |  | .614*** | -.484*** | .904*** |
| 10. Dominant father | -.392*** | -.386*** | .552*** | .762*** | -.394*** | .703*** | -.549*** | -.563*** | .724*** |  | -.500*** | .892*** |
| 11. Paternal warmth | .754*** | .728*** | -.473*** | -.477*** | .751*** | -.506*** | .986*** | .985*** | -.478*** | -.564*** |  | -.547*** |
| 12. Paternal criticism | -.412*** | -.429*** | .684*** | .719*** | -.426*** | .748*** | -.534*** | .-.574*** | .926*** | .930*** | -.562*** |  |

*Note*: Between-person correlations are presented under the diagonal, within-person correlations are presented above the diagonal.

* *p* < .05; ** *p* < .01; *** *p* < .001.

Correlations of parenting during parent-adolescent interactions reported by mothers

|  |  | 2 | 3 | 4 | 5 | 6 |
| --- | --- | --- | --- | --- | --- | --- |
|  |  |  |  |  |  |  |
| 1. Listening |  | .588*** | -.240*** | -.183*** | .866*** | -.234*** |
| 2. Understanding | .847*** |  | -.395*** | -.353*** | .914*** | -.412*** |
| 3. Critical | -.533*** | -.623*** |  | .657*** | -.365*** | .917*** |
| 4. Dominant | -.312*** | -.332*** | .520*** |  | -.311*** | .903*** |
| 5. Parental warmth | .961*** | .961*** | -.602*** | -.335*** |  | -.372*** |
| 6. Parental criticism | -.476*** | -.537*** | .853*** | .889*** | -.527*** |  |

*Note*: Between-person correlations are presented under the diagonal, within-person correlations are presented above the diagonal.

* *p* < .05; ** *p* < .01; *** *p* < .001.

|  |  | 2 | 3 | 4 | 5 | 6 |
| --- | --- | --- | --- | --- | --- | --- |
|  |  |  |  |  |  |  |
| 1. Listening |  | .415*** | -.329*** | -.271*** | .787*** | -.340*** |
| 2. Understanding | .763*** |  | -.373*** | -.309*** | .888*** | -.387*** |
| 3. Critical | -.199 | -.146 |  | .562*** | -.419*** | .890*** |
| 4. Dominant | -.357*** | -.375*** | .618*** |  | -.347*** | .877*** |
| 5. Parental warmth | .935*** | .943*** | -.183 | -.390*** |  | -.434*** |
| 6. Parental criticism | -.308** | -.288** | .902*** | .897*** | -.317** |  |

Correlations of parenting during parent-adolescent interactions reported by fathers

*Note*: Between-person correlations are presented under the diagonal, within-person correlations are presented above the diagonal.

* *p* < .05; ** *p* < .01; *** *p* < .001.

**Appendix 5**

Sensitivity checks parent-adolescent interactions with mothers and fathers.

After finishing data collection, inspection of the data showed that in addition to interactions with mothers (1154 interactions) and fathers (484 interactions) separately, 342 observations concerned interactions with mothers and fathers at the same time. Although the amount of interactions of adolescents with parents on average was close to our expectations described in the preregistration, the number of reported observations with mothers and fathers separately was less than anticipated in the power calculation (minimum of 1200). Therefore, it was decided to include interactions with both parents in the separate analyses regarding parenting of mothers and fathers in relation to adolescent affect. This resulted in a total of 1496 mother-adolescent interactions (*M* = 13.4 per participant, Range = 2-33) based on 112 adolescents and 826 father-adolescent interactions (*M* = 8.98 per participant, Range = 1-30) based on 92 adolescents. Sufficient power is expected for the analyses regarding interactions with mothers, however, power is less regarding interactions with fathers and hence these results should be interpreted with caution.

We performed some sensitivity check for combining data of interactions with mothers and fathers separately and mothers and fathers at the same time. We tested whether positive and negative affect of adolescents differed between interactions with mothers only and mothers and fathers at the same time as well as between interactions with fathers only and mothers and fathers at the same time. Multilevel models indicated that positive and negative affect of adolescents did not differ between interactions with mothers only and mothers and fathers at the same time (all *p*’s > .050). Negative affect of adolescents did not differ between interactions with fathers only and mothers and fathers at the same time (*p* > .050), however positive affect did differ (*p* = .005), indicating that adolescents reported more positive affect during interactions with mothers and fathers compared to only with fathers. Excluding the interactions with mothers and fathers at the same time in analyses regarding interactions with fathers would reduce power, we therefore reran the models regarding interactions with fathers and adolescent positive affect and controlled for interaction with fathers only and mothers and fathers at the same time as additional sensitivity check (Appendix 13).

**Appendix 6**

Correlations of study variables for healthy controls

|  | 1 | 2 | 3 | 4 | 5 | 6 | 7 | 8 |
| --- | --- | --- | --- | --- | --- | --- | --- | --- |
| 1. Positive affect |  | -.537*** | .473*** | -.346*** | .311*** | -.163*** | .234*** | -.197*** |
| 2. Negative affect | -.637*** |  | -.343*** | .528*** | -.252*** | .221** | -.239*** | .246** |
| 3. Positive affect during parent-adolescent interaction | .935*** | -.565*** |  | -.530*** | .433*** | -.274*** | .481*** | -.406** |
| 4. Negative affect during parent-adolescent interaction | -.612*** | .933*** | -.583*** |  | -.359*** | .395*** | -.359*** | .420*** |
| 5. Maternal warmth during parent-adolescent interaction | .635*** | -.442*** | .694*** | -.507*** |  | -.459*** | .573*** | -.360*** |
| 6. Maternal criticism during parent-adolescent interaction | -.346** | .534*** | -.353** | .609*** | -.666*** |  | -.283* | .662*** |
| 7. Paternal warmth during parent-adolescent interaction | .549*** | -.453* | .551*** | -.452** | .685*** | -.394** |  | -.587*** |
| 8. Paternal criticism during parent-adolescent interaction | -.236** | .475*** | -.245** | .532*** | -.440** | .667*** | -.538*** |  |

*Note*: Between-person correlations are presented under the diagonal, within-person correlations are presented above the diagonal.

* *p* < .05; ** *p* < .01; *** *p* < .001.

Correlations of study variables for adolescents with a depression

|  | 1 | 2 | 3 | 4 | 5 | 6 | 7 | 8 |
| --- | --- | --- | --- | --- | --- | --- | --- | --- |
| 1. Positive affect |  | -.591*** | .558*** | -.376*** | .125** | -.103* | .228** | -.128 |
| 2. Negative affect | -.609*** |  | -.427*** | .531*** | -.187*** | .205*** | -.278*** | .192** |
| 3. Positive affect during parent-adolescent interaction | .918*** | -.512** |  | -.619*** | .318*** | -.258*** | .418*** | -.267*** |
| 4. Negative affect during parent-adolescent interaction | -.530** | .878*** | -.538** |  | -.325*** | .388*** | -.302*** | .397*** |
| 5. Maternal warmth during parent-adolescent interaction | .154 | -.026 | .278 | -.108 |  | -.503*** | .848*** | -.271* |
| 6. Maternal criticism during parent-adolescent interaction | -.072 | .325 | -.012 | .284 | -.502** |  | -.161 | .633*** |
| 7. Paternal warmth during parent-adolescent interaction | -.062 | .135 | .074 | .025 | .600** | -.625*** |  | -.416*** |
| 8. Paternal criticism during parent-adolescent interaction | .070 | .141 | .137 | .103 | -.335 | .738*** | -.646*** |  |

*Note*: Between-person correlations are presented under the diagonal, within-person correlations are presented above the diagonal.

* *p* < .05; ** *p* < .01; *** *p* < .001.

**Appendix 7**

Results of models on the association between perceived parenting and adolescent positive affect during parent-adolescent interactions

|  | Maternal warmth |  | Paternal warmth |  | Maternal criticism |  | Paternal criticism |
| --- | --- | --- | --- | --- | --- | --- | --- |
| Fixed effects: estimate *(SE)* |  |  |  |  |  |  |  |
| Intercept | 5.020*** (0.106) |  | 5.086*** (0.124) |  | 5.022*** (0.106) |  | 5.087*** (0.124) |
| Perceived parenting | 0.417*** (0.025) |  | 0.488*** (0.030) |  | -0.266*** (0.025) |  | -0.398*** (0.033) |
| Random effects |  |  |  |  |  |  |  |
| Between-person variance | 1.164 |  | 1.267 |  | 1.153 |  | 1.256 |
| Within-person variance | 0.694 |  | 0.571 |  | 0.769 |  | 0.644 |
|  |  |  |  |  |  |  |  |
| N individuals | 112 |  | 90 |  | 112 |  | 90 |
| N observations | 1491 |  | 818 |  | 1491 |  | 818 |

|  | Maternal warmth |  | Paternal warmth |  | Maternal criticism |  | Paternal criticism |
| --- | --- | --- | --- | --- | --- | --- | --- |
| Fixed effects: estimate *(SE)* |  |  |  |  |  |  |  |
| Intercept | 1.766*** (0.082) |  | 1.729*** (0.088) |  | 1.766*** (0.083) |  | 1.730*** (0.089) |
| Perceived parenting | -0.260*** (0.018) |  | -0.229*** (0.023) |  | 0.279*** (0.017) |  | 0.289*** (0.023) |
| Random effects |  |  |  |  |  |  |  |
| Between-person variance | 0.701 |  | 0.632 |  | 0.711 |  | 0.647 |
| Within-person variance | 0.385 |  | 0.334 |  | 0.364 |  | 0.306 |
|  |  |  |  |  |  |  |  |
| N individuals | 112 |  | 90 |  | 112 |  | 90 |
| N observations | 1491 |  | 818 |  | 1491 |  | 818 |

Results of models on the association between perceived parenting and adolescent negative affect during parent-adolescent interactions

**Appendix 8**


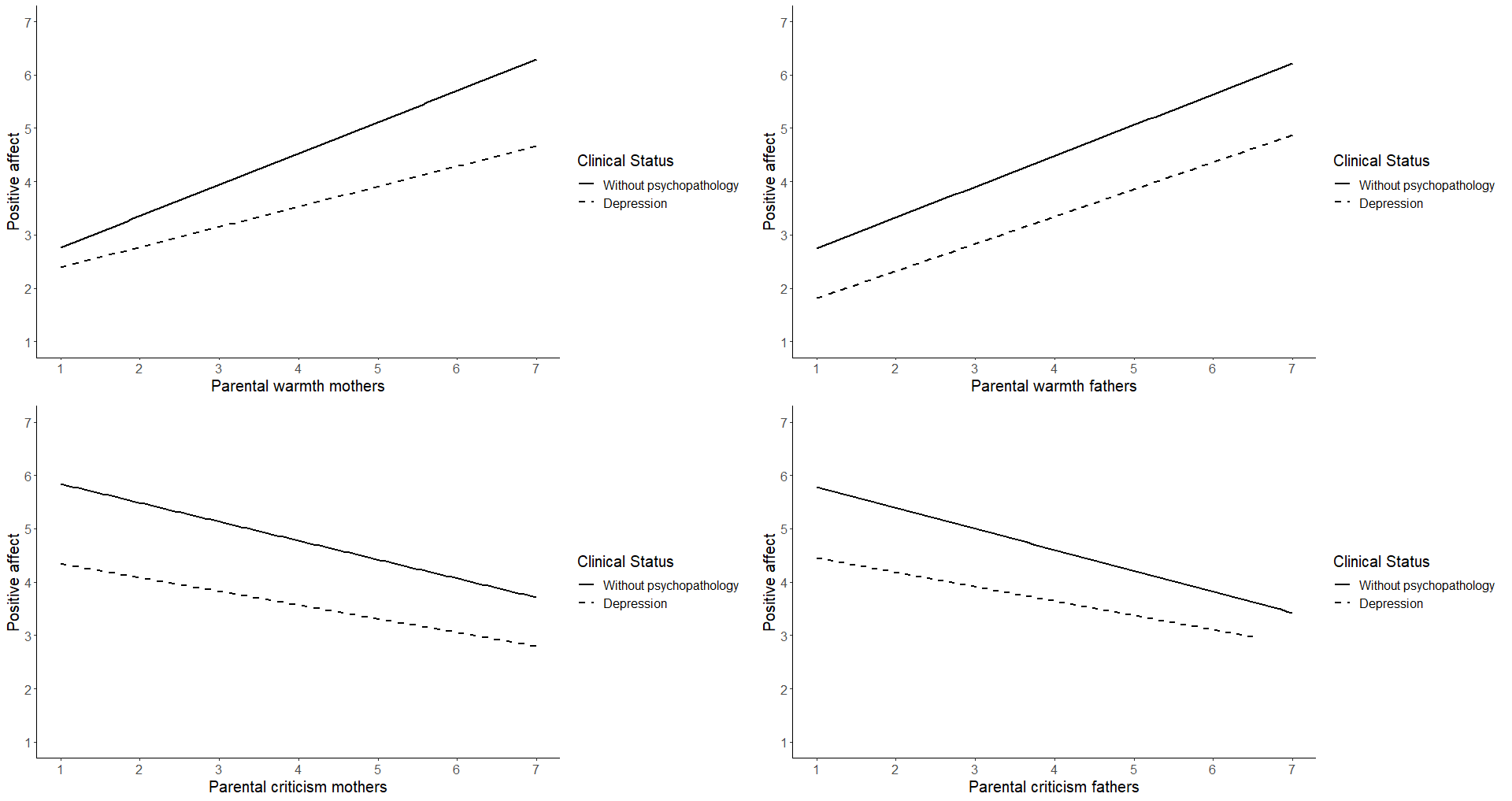


Between-person association between perceived parenting and adolescent positive affect during parent-adolescent interactions. The lines indicate overall association for DEP adolescents and HC adolescents.


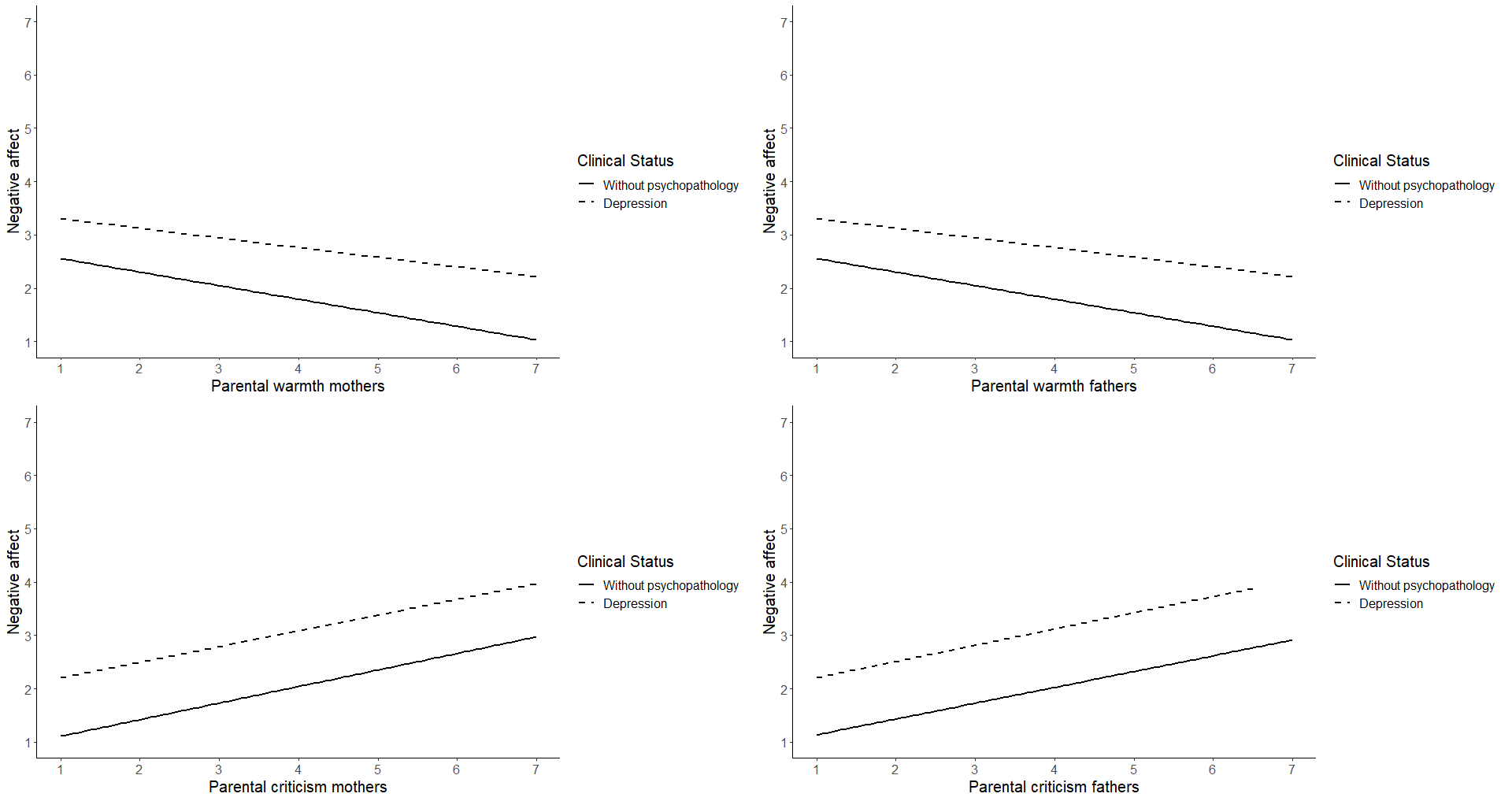


Between-person association between perceived parenting and adolescent negative affect during parent-adolescent interactions. The lines indicate overall association for DEP adolescents and HC adolescents.

**Appendix 9**

Results of models on the relation between perceived parenting and adolescent positive affect during parent-adolescent interactions and the moderating role of depressive symptoms

|  | Maternal warmth |  | Paternal warmth |  | Maternal criticism |  | Paternal criticism |
| --- | --- | --- | --- | --- | --- | --- | --- |
| Fixed effects: estimate *(SE)* |  |  |  |  |  |  |  |
| Intercept | 5.025*** (0.085) |  | 5.012*** (0.100) |  | 5.028*** (0.085) |  | 5.014*** (0.100) |
| Perceived parenting | 0.420*** (0.041) |  | 0.444*** (0.053) |  | -0.269*** (0.040) |  | -0.336*** (0.060) |
| Depressive symptoms (PHQ-9) | -0.084*** (0.011) |  | -0.094*** (0.013) |  | -0.084*** (0.011) |  | -0.094*** (0.013) |
| Perceived parenting*depressive symptoms | -0.004 (0.005) |  | -0.003 (0.007) |  | 0.002 (0.005) |  | 0.004 (0.007) |
| Random effects |  |  |  |  |  |  |  |
| Between-person variance | 0.727 |  | 0.786 |  | 0.715 |  | 0.777 |
| Within-person variance | 0.644 |  | 0.495 |  | 0.724 |  | 0.559 |
| Random effect variance | 0.076 |  | 0.104 |  | 0.066 |  | 0.114 |
|  |  |  |  |  |  |  |  |
| N individuals | 112 |  | 90 |  | 112 |  | 90 |
| N observations | 1491 |  | 818 |  | 1491 |  | 818 |

**p* < .05. ***p* < .01. ****p* < .001.

Results of models on the relation between perceived parenting and adolescent negative affect during parent-adolescent interactions and the moderating role of depressive symptoms

|  | Maternal warmth |  | Paternal warmth |  | Maternal criticism |  | Paternal criticism |
| --- | --- | --- | --- | --- | --- | --- | --- |
| Fixed effects: estimate *(SE)* |  |  |  |  |  |  |  |
| Intercept | 1.760*** (0.059) |  | 1.783*** (0.064) |  | 1.760*** (0.060) |  | 1.783*** (0.064) |
| Perceived parenting | -0.248*** (0.029) |  | -0.186*** (0.040) |  | 0.266*** (0.030) |  | 0.277*** (0.037) |
| Depressive symptoms (PHQ-9) | 0.075*** (0.008) |  | 0.075*** (0.008) |  | 0.075*** (0.008) |  | 0.075*** (0.009) |
| Perceived parenting*depressive symptoms | -0.005 (0.004) |  | 0.002 (0.005) |  | 0.001 (0.004) |  | 0.005 (0.005) |
| Random effects |  |  |  |  |  |  |  |
| Between person variance | 0.337 |  | 0.304 |  | 0.348 |  | 0.315 |
| Within person variance | 0.359 |  | 0.288 |  | 0.334 |  | 0.276 |
| Random effect variance | 0.035 |  | 0.060 |  | 0.044 |  | 0.036 |
|  |  |  |  |  |  |  |  |
| N individuals | 112 |  | 90 |  | 112 |  | 90 |
| N observations | 1491 |  | 818 |  | 1491 |  | 818 |

**p* < .05. ***p* < .01. ****p* < .001.

**Appendix 10**

Results of models on the relation between perceived parenting and adolescent positive affect during parent-adolescent interactions taking into account average perceived parenting

|  | Maternal warmth |  | Paternal warmth |  | Maternal criticism |  | Paternal criticism |
| --- | --- | --- | --- | --- | --- | --- | --- |
| Fixed effects: estimate *(SE)* |  |  |  |  |  |  |  |
| Intercept | 5.377*** (0.077) |  | 5.423*** (0.184) |  | 5.546*** (0.095) |  | 5.442*** (0.115) |
| Daily perceived parenting | 0.417*** (0.041) |  | 0.454*** (0.052) |  | -0.270*** (0.040) |  | -0.344*** (0.060) |
| Average perceived parenting | 0.657*** (0.071) |  | 0.597*** (0.085) |  | -0.370*** (0.087) |  | -0.310** (0.109) |
| Clinical status | -1.205*** (0.141) |  | -1.378*** (0.191) |  | -1.338*** (0.174) |  | -1.422*** (0.229) |
| Random effects |  |  |  |  |  |  |  |
| Between-person variance | 0.375 |  | 0.524 |  | 0.610 |  | 0.784 |
| Within-person variance | 0.644 |  | 0.495 |  | 0.725 |  | 0.556 |
| Random effect variance | 0.078 |  | 0.104 |  | 0.065 |  | 0.118 |
|  |  |  |  |  |  |  |  |
| N individuals | 112 |  | 90 |  | 112 |  | 90 |
| N observations | 1491 |  | 818 |  | 1491 |  | 818 |

**p* < .05. ***p* < .01. ****p* < .001.

Results of models on the relation between perceived parenting and adolescent negative affect during parent-adolescent interactions taking into account average perceived parenting

|  | Maternal warmth |  | Paternal warmth |  | Maternal criticism |  | Paternal criticism |
| --- | --- | --- | --- | --- | --- | --- | --- |
| Fixed effects: estimate *(SE)* |  |  |  |  |  |  |  |
| Intercept | 1.451*** (0.071) |  | 1.446*** (0.071) |  | 1.454*** (0.067) |  | 1.456*** (0.070) |
| Daily perceived parenting | -0.251*** (0.029) |  | -0.192*** (0.040) |  | 0.270*** (0.030) |  | 0.277*** (0.037) |
| Average perceived parenting | -0.304*** (0.066) |  | -0.326*** (0.062) |  | 0.382*** (0.062) |  | 0.359*** (0.067) |
| Clinical status | 1.046*** (0.131) |  | 1.115*** (0.140) |  | 1.037*** (0.123) |  | 1.091*** (0.139) |
| Random effects |  |  |  |  |  |  |  |
| Between-person variance | 0.335 |  | 0.272 |  | 0.302 |  | 0.276 |
| Within-person variance | 0.359 |  | 0.287 |  | 0.334 |  | 0.276 |
| Random effect variance | 0.036 |  | 0.059 |  | 0.043 |  | 0.038 |
|  |  |  |  |  |  |  |  |
| N individuals | 112 |  | 90 |  | 112 |  | 90 |
| N observations | 1491 |  | 818 |  | 1491 |  | 818 |

**p* < .05. ***p* < .01. ****p* < .001.

**Appendix 11**

Results of models on the relation between perceived parenting and adolescent positive affect during parent-adolescent interactions taking into account observation

|  | Maternal warmth |  | Paternal warmth |  | Maternal criticism |  | Paternal criticism |
| --- | --- | --- | --- | --- | --- | --- | --- |
| Fixed effects: estimate *(SE)* |  |  |  |  |  |  |  |
| Intercept | 5.363*** (0.109) |  | 5.332*** (0.129) |  | 5.369*** (0.110) |  | 5.382*** (0.130) |
| Daily perceived parenting | 0.417*** (0.041) |  | 0.442*** (0.053) |  | -0.141*** (0.027) |  | -0.336*** (0.060) |
| Observation | 0.004* (0.002) |  | 0.005**(0.002) |  | 0.004* (0.002) |  | 0.003 (0.002) |
| Clinical status | -1.424*** (0.185) |  | -1.461*** (0.191) |  | -1.4398*** (0.186) |  | -1.469*** (0.238) |
| Random effects |  |  |  |  |  |  |  |
| Between-person variance | 0.725 |  | 0.876 |  | 0.726 |  | 0.862 |
| Within-person variance | 0.641 |  | 0.485 |  | 0.634 |  | 0.555 |
| Random effect variance | 0.078 |  | 0.110 |  | 0.201 |  | 0.117 |
|  |  |  |  |  |  |  |  |
| N individuals | 112 |  | 90 |  | 112 |  | 90 |
| N observations | 1491 |  | 818 |  | 1491 |  | 818 |

**p* < .05. ***p* < .01. ****p* < .001.

Results of models on the relation between perceived parenting and adolescent negative affect during parent-adolescent interactions taking into account observation

|  | Maternal warmth |  | Paternal warmth |  | Maternal criticism |  | Paternal criticism |
| --- | --- | --- | --- | --- | --- | --- | --- |
| Fixed effects: estimate *(SE)* |  |  |  |  |  |  |  |
| Intercept | 1.390*** (0.083) |  | 1.459*** (0.088) |  | 1.390*** (0.083) |  | 1.430*** (0.087) |
| Daily perceived parenting | -0.248*** (0.029) |  | -0.188*** (0.040) |  | 0.268*** (0.030) |  | 0.276*** (0.037) |
| Observation | 0.001 (0.001) |  | -0.001 (0.001) |  | 0.001 (0.001) |  | -0.000 (0.001) |
| Clinical status | 1.143*** (0.140) |  | 1.159*** (0.158) |  | 1.149*** (0.141) |  | 1.160*** (0.159) |
| Random effects |  |  |  |  |  |  |  |
| Between-person variance | 0.408 |  | 0.366 |  | 0.3419 |  | 0.378 |
| Within-person variance | 0.359 |  | 0.287 |  | 0.333 |  | 0.276 |
| Random effect variance | 0.037 |  | 0.061 |  | 0.043 |  | 0.038 |
|  |  |  |  |  |  |  |  |
| N individuals | 112 |  | 90 |  | 112 |  | 90 |
| N observations | 1491 |  | 818 |  | 1491 |  | 818 |

**p* < .05. ***p* < .01. ****p* < .001.

**Appendix 12**

Results of models on the relation between perceived parenting and adolescent positive affect during parent-adolescent interactions and the moderating role of sex

|  | Maternal warmth |  | Paternal warmth |  | Maternal criticism |  | Paternal criticism |
| --- | --- | --- | --- | --- | --- | --- | --- |
| Fixed effects: estimate *(SE)* |  |  |  |  |  |  |  |
| Intercept | 5.653*** (0.153) |  | 5.865*** (0.184) |  | 5.663*** (0.153) |  | 5.866*** (0.184) |
| Perceived parenting | 0.338*** (0.072) |  | 0.433*** (0.096) |  | -0.260*** (0.066) |  | -0.271** (0.102) |
| Sex | -0.315 (0.181) |  | -0.612** (0.219) |  | -0.315 (0.181) |  | -0.609** (0.219) |
| Clinical status | -1.377*** (0.183) |  | -1.373*** (0.231) |  | -1.402*** (0.184) |  | -1.379*** (0.231) |
| Perceived parenting*sex | 0.121 (0.087) |  | 0.023 (0.115) |  | -0.011 (0.083) |  | -0.111 (0.126) |
| Random effects |  |  |  |  |  |  |  |
| Between-person variance | 0.704 |  | 0.791 |  | 0.692 |  | 0.783 |
| Within-person variance | 0.644 |  | 0.495 |  | 0.724 |  | 0.558 |
| Random effect variance | 0.075 |  | 0.103 |  | 0.065 |  | 0.115 |
|  |  |  |  |  |  |  |  |
| N individuals | 112 |  | 90 |  | 112 |  | 90 |
| N observations | 1491 |  | 818 |  | 1491 |  | 818 |

**p* < .05. ***p* < .01. ****p* < .001.

Results of models on the relation between perceived parenting and adolescent negative affect during parent-adolescent interactions and the moderating role of sex

|  | Maternal warmth |  | Paternal warmth |  | Maternal criticism |  | Paternal criticism |
| --- | --- | --- | --- | --- | --- | --- | --- |
| Fixed effects: estimate *(SE)* |  |  |  |  |  |  |  |
| Intercept | 1.341*** (0.117) |  | 1.203*** (0.124) |  | 1.340*** (0.117) |  | 1.201*** (0.125) |
| Perceived parenting | -0.206*** (0.051) |  | -0.203** (0.073) |  | 0.241*** (0.049) |  | 0.235** (0.064) |
| Sex | 0.117 (0.138) |  | 0.340* (0.147) |  | 0.117 (0.139) |  | 0.340* (0.149) |
| Clinical status | 1.132*** (0.141) |  | 1.106*** (0.155) |  | 1.137*** (0.141) |  | 1.117*** (0.157) |
| Perceived parenting*sex | -0.062 (0.062) |  | 0.020 (0.087) |  | 0.044 (0.062) |  | 0.068 (0.078) |
| Random effects |  |  |  |  |  |  |  |
| Between-person variance | 0.404 |  | 0.343 |  | 0.415 |  | 0.357 |
| Within-person variance | 0.359 |  | 0.288 |  | 0.333 |  | 0.276 |
| Random effect variance | 0.037 |  | 0.060 |  | 0.043 |  | 0.038 |
|  |  |  |  |  |  |  |  |
| N individuals | 112 |  | 90 |  | 112 |  | 90 |
| N observations | 1491 |  | 818 |  | 1491 |  | 818 |

**p* < .05. ***p* < .01. ****p* < .001.

**Appendix 13**

We reran the models regarding interactions with fathers and adolescent positive affect and added a dichotomous variable indicating whether the interaction was with fathers only (0) or mothers and fathers at the same time (1). Results of the previous models did not change and additionally it was found that adolescents reported more positive affect during interactions with mothers and fathers at the same time compared to during interactions only with fathers in the models including parental warmth and criticism of fathers. See below for full model results.

Results of models on the relation between parental warmth of fathers and adolescent positive affect during parent-adolescent interactions controlled for interacting with fathers only or mothers and fathers at the same time

|  | Paternal warmth | | | | | | |
| --- | --- | --- | --- | --- | --- | --- | --- |
| Fixed effects: estimate *(SE)* |  |  |  |  |  |  |  |
| Intercept | 5.021*** (0.127) |  | 5.412*** (0.123) |  | 4.961*** (0.103) |  | 5.817*** (0.187) |
| Perceived parenting | 0.484*** (0.030) |  | 0.447*** (0.060) |  | 0.441*** (0.052) |  | 0.430*** (0.095) |
| Clinical status |  |  | -1.469*** (0.240) |  |  |  | -1.370*** (0.233) |
| Depressive symptoms (PHQ-9) |  |  |  |  | -0.094*** (0.013) |  |  |
| Sex |  |  |  |  |  |  | -0.617** (0.221) |
| Father vs both parents | 0.169** (0.064) |  | 0.132* (0.062) |  | 0.134* (0.061) |  | 0.133* (0.061) |
| Perceived parenting*clinical status |  |  | -0.001 (0.120) |  |  |  |  |
| Perceived parenting*depressive symptoms |  |  |  |  | -0.003 (0.007) |  |  |
| Perceived parenting*sex |  |  |  |  |  |  | 0.024 (0.113) |
| Random effects |  |  |  |  |  |  |  |
| Between-person variance | 1.279 |  | 0.877 |  | 0.795 |  | 0.800 |
| Within-person variance | 0.569 |  | 0.494 |  | 0.493 |  | 0.494 |
| Random effect variance |  |  | 0.099 |  | 0.100 |  | 0.099 |
|  |  |  |  |  |  |  |  |
| N individuals | 90 |  | 90 |  | 90 |  | 90 |
| N observations | 818 |  | 818 |  | 818 |  | 818 |

Results of models on the relation between parental criticism of fathers and adolescent positive affect during parent-adolescent interactions controlled for interacting with fathers only or mothers and fathers at the same time

|  | Paternal criticism | | | | | | |
| --- | --- | --- | --- | --- | --- | --- | --- |
| Fixed effects: estimate *(SE)* |  |  |  |  |  |  |  |
| Intercept | 5.004*** (0.128) |  | 5.381*** (0.124) |  | 4.930*** (0.104) |  | 5.787*** (0.188) |
| Perceived parenting | -0.398*** (0.033) |  | -0.357*** (0.069) |  | -0.336*** (0.059) |  | -0.270** (0.101) |
| Clinical status |  |  | -1.467*** (0.241) |  |  |  | -1.375*** (0.234) |
| Depressive symptoms (PHQ-9) |  |  |  |  | -0.094*** (0.013) |  |  |
| Sex |  |  |  |  |  |  | -0.617** (0.222) |
| Father vs both parents | 0.217** (0.068) |  | 0.218*** (0.065) |  | 0.219*** (0.065) |  | 0.216*** (0.065) |
| Perceived parenting*clinical status |  |  | 0.065 (0.138) |  |  |  |  |
| Perceived parenting*depressive symptoms |  |  |  |  | 0.005 (0.007) |  |  |
| Perceived parenting*sex |  |  |  |  |  |  | -0.114 (0.125) |
| Random effects |  |  |  |  |  |  |  |
| Between-person variance | 1.275 |  | 0.878 |  | 0.794 |  | 0.800 |
| Within-person variance | 0.636 |  | 0.551 |  | 0.551 |  | 0.551 |
| Random effect variance |  |  | 0.116 |  | 0.112 |  | 0.114 |
|  |  |  |  |  |  |  |  |
| N individuals | 90 |  | 90 |  | 90 |  | 90 |
| N observations | 818 |  | 818 |  | 818 |  | 818 |
